# A bibliometric analysis of global health curriculum teaching models: Current status, hotspots, and trends in higher education between 2014 and 2024

**DOI:** 10.1016/j.ghrp.2026.04.004

**Published:** 2026-04-27

**Authors:** Beiran Qian, Yuxuan Li, Bin Wu, Yutong Lu, Kun Tang

**Affiliations:** a Vanke School of Public Health, Tsinghua University, Beijing, China; b School of Basic Medical Sciences, Tsinghua University, Beijing, China; c School of Public Health, Imperial College London, London, United Kingdom; d School of Nursing, Chinese Academy of Medical Sciences & Peking Union Medical College, Beijing, China

**Keywords:** Global Health Education, Disciplinary Development, Competency-based Education, Interprofessional Education, Bibliometric

## Abstract

**Background:**

As global health challenges become increasingly complex, understanding the dynamic evolution of teaching models in higher education is critical. This study aims to employ bibliometric methods to map the status, identify hotpots, and capture key trends in global health curriculum design over the past decade.

**Methods:**

Utilizing CiteSpace 6.4.R1, a comprehensive bibliometric analysis was conducted on 209 studies focusing on global health curriculum teaching models, indexed in the Web of Science Core Collection between 2014 and 2024. Articles and reviews published in English were retrieved using a structured search strategy combining four keyword domains: “Global Health”, “Curriculum”, “Teaching Model”, and “Higher Education”. The analysis comprised performance analysis and science mapping techniques, including co-authorship analysis, Global North-South collaboration assessment, co-citation analysis, keyword co-occurrence and clustering, and burst detection to identify emerging research fronts.

**Results:**

Global health education has experienced rapid development over the past decade, with the number of publications increasing from 9 in 2014 to 38 in 2020, and maintaining a relatively high level since then. Developed countries, particularly the United States, are leading the field in terms of publication volume (n = 121, 57.9%), while Global South institution’s participation remained limited (28.9%). North-South collaborations were uneven, with 77.6% of curricula incorporating collaborative development elements but only 57.9% reflecting co-authorship partnerships in transnational studies. According to the co-occurrence analyses of keywords, the most frequently occurring teaching models are “Interprofessional learning” (n = 9), “Competency-based education” (n = 8), “Online learning” (n = 7), “Experiential learning” (n = 4) and “Community-engaged learning” (n = 4).

**Conclusion:**

This study provides a comprehensive evaluation of global health curriculum teaching models, highlighting key developments and trends. The field has advanced markedly but reflects persistent inequities in knowledge production and resource allocation. Future efforts must prioritize equitable North-South collaboration, culturally adapted competency frameworks, and inclusive technological integration to foster a globally representative workforce capable of addressing interconnected health challenges. Moving forward, global health education should embrace dynamic, forward-thinking approaches to prepare a versatile workforce capable of tackling both current and future global health challenges.

## Highlights

• Global health teaching research increased with developed nations leading the field.
• Competency-based education has become a core strategy for global health teaching.
• Interprofessional learning fosters critical interdisciplinary skills for students.
• Global health events and technology have driven the adoption of virtual education.
• Equity-focused collaboration must reshape global health education development.

## Introduction

Global health is a critical and rapidly growing field, confronting urgent public health challenges, climate change, and prolonged conflicts. Global health is defined as an area for study, research, and practice that places a priority on improving health and achieving equity in health for all people worldwide.^1^ Evolving from international health, it emphasizes transnational health issues, determinants, and solutions; engages numerous disciplines within and beyond the health sciences and promotes interdisciplinary collaboration; and integrates population-based prevention with individual-level clinical care.^2^ In this context, advancing global health education is critical to providing students with a comprehensive and nuanced understanding of the multifaceted nature of global health and realizing the vision of “health for all”.

Global health education has evolved unevenly across regions while simultaneously expanding in scope and pedagogical innovation. In high-income countries (HICs), tertiary institutions have long played a leading role, beginning with Johns Hopkins University’s launch of its first international health program in 1961, which laid the foundation for the institutionalization of global health education.^3^ Since then, these programs have expanded to include a wide range of course formats, such as electives, degree programs, and integration with medical curricula, broadening access to more students.^4^ Course content also varies geographically, reflecting the field’s interdisciplinary nature: universities in the United States, such as Harvard, provide wide-ranging thematic coverage,^5^ whereas institutions in the United Kingdom, including the London School of Hygiene & Tropical Medicine, emphasize social science perspectives.^6^ In contrast, significant disparities persist between the Global North and South. Institutions from High-Income and Upper-Middle-Income countries constitute 88.6% of the Consortium of Universities for Global Health (CUGH) membership,^7^ underscoring structural imbalances in educational capacity. Economic and geographic constraints in many low- and middle- income countries (LMICs) limit the training of professionals with competencies in cross-border health governance, health systems strengthening, and international disease control, while curricula may insufficiently align with local priorities.^4^ Meanwhile, global health education has undergone substantive content and methodological transformation. Multidisciplinary teaching models have cultivated professionals capable of addressing complex health determinants,^8^, ^9^ and the rapid expansion of online learning has reduced geographic barriers.^10^, ^11^, ^12^ Thematic frontiers have broadened to incorporate “One Health”, “Climate Change”, and “Planetary Health”, signaling a shift toward recognizing the interconnected ecological, social, and political dimensions of health. Complementing these developments, key frameworks including the Global Health Education Framework,^13^ the 5Ps (parity, people, planet, priorities, and practices) Global Health Education Model,^14^ and the Interactive Framework for Decolonizing Global Health Education^15^ have provided structured yet context-sensitive guidance for curriculum design, ethical engagement, and competency development.

Considering the growing significance of global health and ongoing structural inequities in knowledge generation and resource allocation, a comprehensive exploration of global health education design is essential for structuring curricula that effectively address multifaceted health issues. Existing research is predominantly concentrated in HICs, reflecting their earlier and more rapid institutional development in this field. Much of the literature focuses on single-country experiences, shaped both by variations in national education systems and by scholars’ emphasis on domestic reform agendas.^6^, ^16^, ^17^ Although some global reviews have been conducted,^16^ they lack comprehensive synthesis and critical examination of pedagogical models, particularly from the perspective of regional inequities and Global North–South collaboration.^18^ Moreover, the current evidence base is largely descriptive and cross-sectional,^19^, ^20^ with limited longitudinal evaluation of how curricula evolve in response to shifting global health priorities. This constrains a deeper understanding of structural imbalances and the dynamic transformation of global health education.

To address these gaps, this study conducts a bibliometric analysis of global health education literature published over the past decade. It seeks to answer one overarching question: Over the past decade from 2014 to 2024, what are the development patterns of global health education research and what pedagogical priorities and future trajectories characterize this field? By systematically mapping publication trends, thematic domains, and collaboration networks, the study traces the evolution of teaching models in response to emerging global health challenges while identifying research frontiers, technological innovations, and persistent unmet needs. By employing bibliometric methods, this study aims to strengthen cross-regional understanding, inform the development of more structured and globally representative curricula, and promote more equitable educational partnerships and resource allocation in global health education.

## Methods

This study adopts a bibliometric approach to address our research questions and provide a systematic, evidence-based overview of the field's evolution over the past decade. As a modern branch of scientometrics, bibliometric analysis applies mathematical and statistical methods to the examination of scientific publications. This methodology involves the use of quantitative techniques to process bibliometric data, enabling researchers to map the current landscape and uncover emerging trends within a specific field.^21^

### Database selection

The Web of Science Core Collection (WoSCC) was selected as the data source for this bibliometric study. The choice of WoSCC was based on three principal considerations. First, WoSCC encompasses three major indexes, including the Science Citation Index Expanded, Social Sciences Citation Index, and Arts & Humanities Citation Index, which make it an ideal resource for in-depth analysis of specialized fields across the natural sciences, social sciences, and humanities, all of which are relevant to global health education research.^22^ Second, WoSCC provides advanced search features with an extensive range of tools, and is internationally recognized for its high quality standards, enabling the construction of reproducible and comprehensive search strategies with Boolean operators and field-specific queries, thereby making it most widely accepted for scientific publication analysis.^23^ Third, WoSCC features built-in data export functions and reference format fully compatible with CiteSpace, the bibliometric visualization tool used in this study, thereby facilitating seamless data processing.^24^

### Search strategy

To comprehensively capture the literature on global health curriculum teaching models, a structured search strategy was developed using four keyword domains: (1) “Global Health”, incorporating related terms such as “international health”; (2) “Curriculum”, encompassing expressions including “course”, “lesson”, and “teaching program”, as well as different course types such as electives and compulsory courses; (3) “Teaching Model”, covering related concepts such as “curriculum design”, “curriculum development”, “educational framework”, and “teaching pedagogy”; and (4) “Higher Education”. Boolean operators were applied to construct the final query: “OR” was used within each keyword domain to broaden the search, while “AND” was applied across the four domains to ensure all key dimensions were captured simultaneously. The complete search strategy is shown in Table 1.

**Table 1:** Search Strategy in Web of Science Core Collection.

**Step**	**Search Format**	**Results**
#1	TS= ("global health" OR "international health" OR "world health")	107,232
#2	TS= ("course*" OR "curricul*" OR "lesson*" OR "teach*" OR "educat*" OR "learn*" OR "class*" OR "elective*" OR "compulsor*")	3941,279
#3	TS= ("model*" OR "design*" OR "pedagog*" OR "strateg*" OR "develop*" OR "framework*" OR "approach*" OR "implement*" OR "method*" OR "innovat*")	17,076,443
#4	TS= ("higher education" OR "universit*" OR "college*" OR "tertiary education" OR "graduate*" OR "undergraduate*")	721,747
#5	#1 AND #2 AND #3 AND #4	3331

Note: Search date was from 1st January 2014 to 14th November 2024.

### Search refinement

To ensure the precision and relevance of the retrieved literature, the initial search results were further refined according to the following criteria. Document type was restricted to “article” and “review”, excluding book chapters, conference proceedings, editorials, and letters, in order to focus on substantive, peer-reviewed contributions. The publication language was limited to English to ensure consistency in data extraction and analysis. The study period spanned from 2014 to 2024, a ten-year window that captures the modern trajectory of global health curriculum development while maintaining a focused and manageable dataset. Following these refinements, the retrieved results were sorted by relevance, and the top 100 most-cited records were manually reviewed by two independent researchers to confirm alignment with the research scope and to verify the true-positive results.

### Inclusion and exclusion criteria

Studies were eligible for inclusion if they focused on specific global health curricula, courses, or teaching programs implemented in higher education institutions. For consistency, the term “curriculum” is used throughout this manuscript as an umbrella term encompassing all such formats. Studies were excluded if they: (a) mentioned global health education only in passing without specifically addressing curricula, courses, or pedagogical frameworks; (b) were conducted in settings outside higher education, including secondary schools, hospitals, or primary healthcare institutions; or (c) represented duplicate publications or updated versions of previously included studies.

### Data extraction and pre-processing

**Fig. 1:**
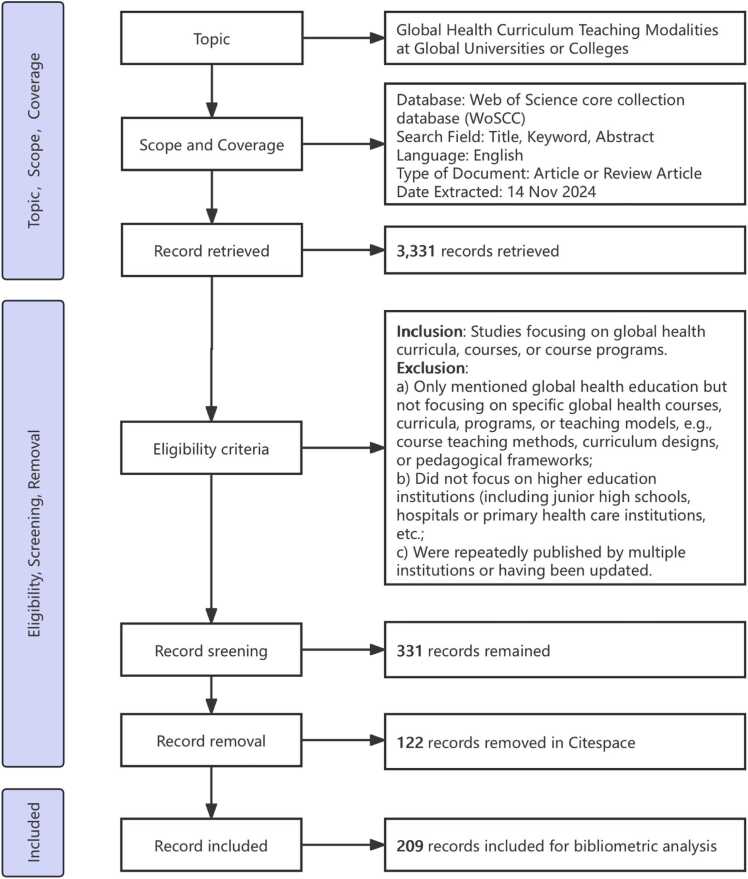
Flow chart of data extraction and pre-processing.

Initially, 3331 studies were retrieved for screening. Data extraction was independently carried out by two researchers, who searched, downloaded, and validated the articles, resolving discrepancies through discussion to ensure standardized results. After screening against the inclusion and exclusion criteria, 331 studies remained and were downloaded in plain text format. These were imported into CiteSpace 6.4.R1, where data pre-processing primarily consisted of filtering and removal of duplicate records (n = 122). A final set of 209 studies was retained for bibliometric analysis (Fig. 1).

### Data analysis

Within CiteSpace 6.4.R1, the time span was set from January 2014 to December 2024, with a time slice of one year per slice. The scaling factor k was set to 25. Network pruning was performed using the Pathfinder algorithm, with both “Pruning Sliced Networks” and “Pruning the Merged Network” options enabled to reduce noise and enhance the clarity of the resulting maps. In all visualizations, node size is proportional to the frequency of occurrence, with larger nodes indicating higher prominence.

Annual publication volume was calculated to capture temporal trends in the field over the study period. Node type “Country” was selected to construct co-occurrence networks, enabling identification of the most productive countries and their collaboration patterns. Node type “Institution” was used to analyze institutional contributions and identify leading organizations in global health curriculum research. Node types “Reference” and “Cited Journal” were configured to perform co-citation analyses, allowing identification of the most authoritative and frequently referenced sources in the field. Author Keywords (DE) were selected for keyword co-occurrence analysis to identify major research themes.

In keyword cluster analysis, cluster labels and representative terms were algorithmically generated by CiteSpace, providing an approximate categorization rather than a definitive taxonomy. Clusters were numbered in descending order of size, starting from the largest (#0), followed by #1, #2, and so on. For keyword burst analysis, the gamma parameter was set to 1.5 and the minimum burst duration was set to 2 years by default. Burst strength reflects the relative surge in keyword frequency within a given period. In the visualization, red segments indicate periods of peak burst intensity, while blue segments represent sustained but lower-level scholarly attention.

Global North-South collaboration was assessed across two dimensions. First, authorship collaboration refers to co-authorship relationships between institutions affiliated with the Global North (developed countries) and the Global South (developing countries).^25^ Second, collaboration in curriculum development encompasses joint efforts in the design of educational content, pedagogical approaches, and assessment frameworks. Studies that focused solely on a single country’s global health education, without involving international co-authorship, were excluded from the Global North-South collaboration analysis. Additionally, interprofessional collaboration in authorship is defined as interprofessional/interdisciplinary research collaboration that occurs when researchers from more than one profession or discipline work together to achieve the common goal of producing new scientific knowledge.^26^

### Bibliometric indicators

The bibliometric analysis in this study was conducted across six methodologically standardized stages, consistent with the analytical framework re--ed for bibliometric research. (1) Annual publication volume was calculated to capture temporal trends in the field. (2) Co-occurring national analysis was performed to identify the most productive countries. (3) Co-occurring institutional analysis was conducted to determine the leading contributing organizations. (4) Co-citation analysis of journals and literature was used to identify the most authoritative and frequently referenced sources in the field. (5) Keyword co-occurrence analysis, keyword cluster interpretation, and keyword burst detection were applied to map research hotspots and emerging trends in global health curriculum teaching models. (6) Global North–South collaboration was assessed across two dimensions: co-authorship patterns between Global North and Global South institutions, and North–South partnerships in curriculum development. Additionally, interprofessional collaboration in authorship was analyzed to explore cross-disciplinary engagement in global health education research.

## Results

### Characteristics of the retrieved publications

The included 209 studies were primarily published as research articles (n = 191; 91.4%) and review articles (n = 18; 8.6%). In terms of study focus, 133 studies (63.6%) concentrated exclusively on domestic education systems within a single country, while 76 studies (36.4%) examined cross-national global health education.

**Fig. 2:**
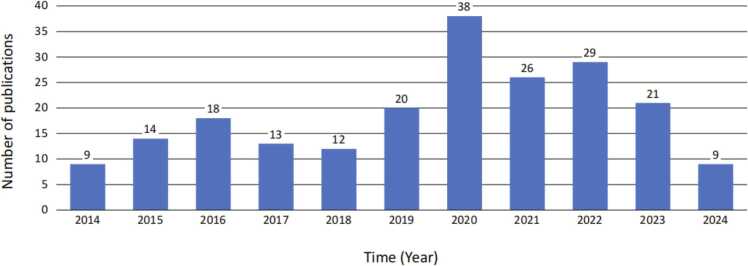
The number of annual publications between 2014 and 2024.

Regarding annual publication trends, the overall volume showed an upward trajectory over the study period. Prior to 2019, the annual number of publications remained relatively stable. A marked surge occurred in 2020, with the number of publications approximately doubling compared to 2019, coinciding with the onset of the COVID-19 pandemic. Output remained elevated through 2021 and 2022, before beginning a gradual decline in 2023 (Fig. 2).

### Active countries

Co-occurring national analysis was conducted to identify the most productive countries in the field of global health curriculum teaching models. Table 2 presents the top five countries in terms of publication frequency. The United States ranked first with 121 publications, accounting for 57.9% of the total, reflecting its long-established leadership in global health education. Canada, England, Australia, and Germany also demonstrated notable research activity in this field.

**Table 2:** Top 5 Countries and Institutions with the Most Publications.

**Rank**	**Country (Frequency, %)**	**Institution (Frequency, Country of Affiliation)**
1	The United States (121, 57.9%)	Harvard University (25, The United States)
2	Canada (26, 12.4%)	Johns Hopkins University (20, The United States)
3	England (17, 12.4%)	University of California System (14, The United States)
4	Australia (13, 6.2%)	Ohio State University (7, The United States)
5	Germany (12, 5.7%)	Duke University (7, The United States)

Note: Multiple occurrences of a country/an institution of co-authors in the same paper are counted once.

### Active institutions

The top five contributing institutions were all based in the United States, with Harvard University (n = 25) and Johns Hopkins University (n = 20) leading in terms of publication volume, followed by University of California System (n = 14), Ohio State University (n = 7) and Duke University (n = 7) (Table 2). Among included studies, institutions from Global South countries accounted for only 28.9% (105/364) of total institutional participation.

### Active journals

**Fig. 3:**
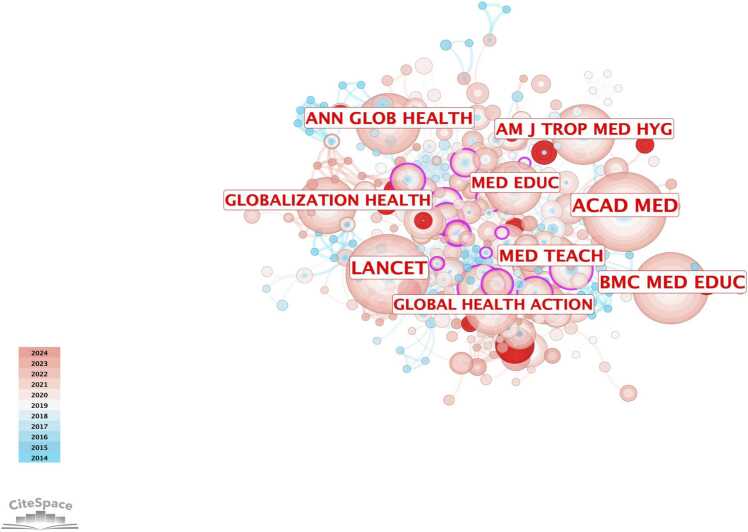
**Co-occurrence Map of Co-cited Journal.** Top 5 journal with the most citation were ACAD MED (Academic Medicine), LANCET, BMC MED EDUC (BMC Medical Education), ANN GLOB HEALTH (Annals of Global Health) and MED TEACH (Medical Teacher).

Co-citation analysis of journals was performed to identify the most authoritative and frequently referenced publication outlets in the field of global health teaching models. Academic Medicine was the most co-cited journal (n = 120), followed closely by Lancet (n = 119), BMC Medical Education (n = 100), Annals of Global Health (n = 75), and Medical Teacher (n = 69), constituting the top five most cited journals (Fig. 3). The prominence of education-focused medical journals alongside high-impact general health journals reflects the interdisciplinary nature of global health curriculum research.

### Top 10 co-cited literature

The top 10 most frequently co-cited studies are listed in Appendix Table 1 based on citation frequency across the 209 included publications. These highly cited works were primarily published between 2012 and 2020, with the highest concentration occurring in the mid-2010s. The most frequently co-cited article was *Identifying Inter-professional Global Health Competencies for 21st-Century Health Professionals*, published in Annals of Global Health in 2015, which describes the CUGH Subcommittee’s work on defining interprofessional global health competencies and has been widely adopted as a reference standard in the design of global health curricula and training programs.

### Top 10 teaching models

**Fig. 4:**
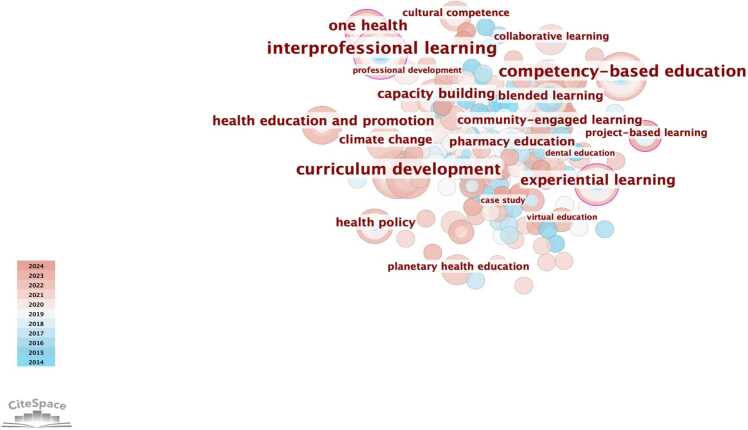
Co-occurrence Map of Keywords.

Keyword co-occurrence analysis was applied to identify the dominant teaching models represented in the retrieved literature. Table 3 lists the top 10 teaching model keywords by frequency of occurrence **(**Fig. 4**)**. “Interprofessional learning” ranked first with the highest frequency (n = 9), reflecting a strong emphasis on cross-disciplinary collaboration in global health education. “Competency-based education” (n = 8) followed, highlighting a shift towards outcome-driven curricula aimed at equipping learners with essential skills. “Online learning” (n = 7) emerged as a prominent model, likely driven by the rapid adoption of digital platforms during the COVID-19 pandemic.

**Table 3:** Top 10 Keywords of Teaching Models and Topics.

**Rank**	**Keywords of Teaching Models**	**Frequency**	**Keywords of Teaching Topics**	**Frequency**
1	Interprofessional learning	n = 9	One Health	n = 6
2	Competency-based education	n = 8	Health education and promotion	n = 5
3	Online learning	n = 7	Climate change	n = 4
4	Experiential learning	n = 4	Health policy	n = 4
5	Community-engaged learning	n = 4	Health equity	n = 3
6	Blended learning	n = 3	Planetary health education	n = 3
7	Project-based learning	n = 3	Population health	n = 3
8	Collaborative learning	n = 2	Global health diplomacy	n = 2
9	Virtual education	n = 2	Covid-19 pandemic	n = 2
10	Cross-cultural learning and teaching	n = 2	Health systems	n = 2

### Top 10 teaching topics

The co-occurrence analysis further revealed the dominant thematic topics addressed in global health curricula. Table 3 also presents the top 10 teaching topic keywords. “One Health” (n = 6) was the most frequently discussed topic, reflecting growing recognition of the interconnectedness of human, animal, and environmental health in global health education. It was followed closely by “Health education and promotion” (n = 5), “Climate change” (n = 4), and “Health policy” (n = 4), highlighting the expanding thematic scope of global health curricula beyond traditional biomedical frameworks **(**Fig. 4**)**.

### Keyword clusters

**Fig. 5:**
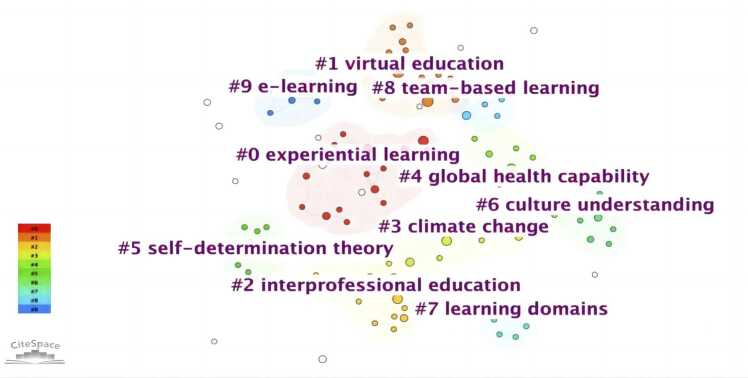
Co-occurrence Map of Keywords Cluster Domains.

Keyword cluster analysis was conducted to identify underlying thematic groupings within the co-occurrence network. Using CiteSpace’s likelihood ratio algorithm, the largest k connected components were extracted, generating ten distinct clusters from the keyword co-occurrence network. These clusters include “Experiential learning”, “Virtual education”, “Interprofessional education”, “Climate change”, among others (Fig. 5). Table 4 presents the main keywords associated with each cluster, revealing their respective thematic focus.

**Table 4:** Keywords Cluster Domains and Main Keywords.

**Cluster**	**Label**	**Main Keywords**
#0	Experiential learning	Experiential learning, health education and promotion, project-based learning, cultural competence, health policy, disease prevention, control strategies
#1	Virtual education	Virtual education, curriculum development, competence-based education, professional development, change management, didactic learning
#2	Interprofessional education	Interprofessional learning, collaborative learning, case study, One Health, institutional partnership
#3	Climate change	Climate change, blended learning, planetary health education, environmental health, health system research, curriculum integration
#4	Global health capability	Online learning, covid-19 pandemic, critical thinking, global citizenship, clinical skills, curriculum design, global health capability
#5	Self-determination theory	Self-determination theory, community-engaged learning, student autonomy, medical service learning, reflective journals, public health work
#6	Culture understanding	Culture understanding, global health nursing, global health competencies, education intervention
#7	Learning domains	Instrument development, programme evaluation, host country partnership
#8	Team-based learning	Team-based learning, electives, pharmacy education, education assessment, comparative health delivery system
#9	E-learning	Cross-cultural learning and teaching, peer learning, constructivist learning approach

Note: # refers to the serial number of the clusters. The clusters are numbered in the descending order of the cluster size, starting from the largest cluster #0, the second largest #1, and so on.

### Burst keywords

**Fig. 6:**
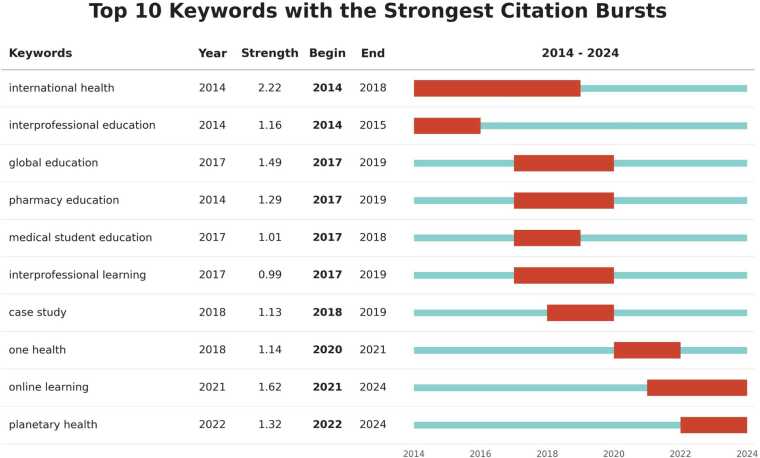
Top 10 Keywords with the Strongest Citation Burst.

The top 10 keywords with the strongest citation bursts in global health education teaching models from 2014 to 2024 are presented in Fig. 6. From 2014, “international health” and “interprofessional education” showed strong bursts. During 2017 to 2018, keywords including “global education”, “pharmacy education”, “medical student education”, “interprofessional learning” and “case study” emerged with burst activity. From 2020 onward, “one health”, “online learning” and “planetary health” demonstrated citation bursts.

### Global North-South collaboration

**Fig. 7:**
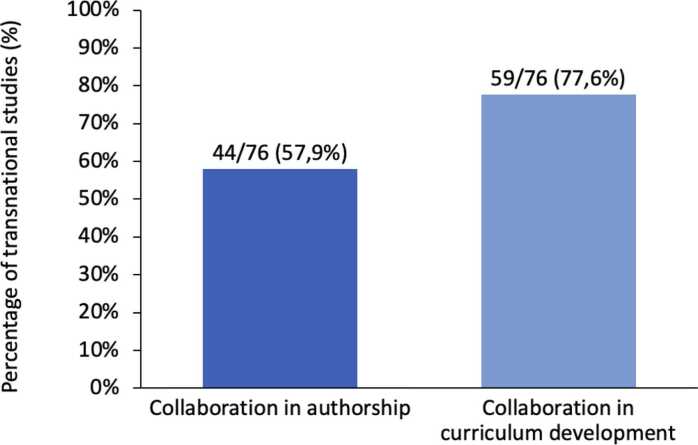
**Collaboration patterns of global health education studies**. (a) Distribution of studies by scope (N = 209);(b) Global North-South collaboration within transnational studies (N = 76).

Among the 76 studies involving cross-national global health education, 77.6% (59/76) reported Global North–South collaboration in curriculum design, whereas only 57.9% (44/76) demonstrated North–South co-authorship (Fig. 7). Collaborative curriculum formats included seminars (n = 4), practice programs (n = 21), teaching programs (n = 31), and other types (n = 21) (Table 5). International health electives where medical students from HICs undertake both teaching programs and field practice in LMICs, represented a common model of North–South collaboration. Across all 209 studies, 146 (69.8%) incorporated interprofessional collaboration in authorship (Appendix Table 2).

**Table 5:** Category of Curricula Development in Global North-South Collaboration.

Category	Examples of Collaboration	Studies
Seminars	North-South Institutional Collaborative Seminars	n = 4
Teaching programs	North-South University Joint Education Programs/Courses, North-South Student Collaborative Learning, North-South Faculty Team Teaching, etc.	n = 31
Practice programs	International Field Practice Collaborations, Case-Based Programme, etc.	n = 21
Other types	Model WHA Programs, Model WHO Programs, Joint Research Projects, Partnership Programs, etc.	n = 21

Note: One study may involve more than one category of curricula development.

## Discussion

This study employs bibliometric methods to comprehensively assess the current landscape, key focus areas, and emerging trends in global health curriculum teaching models. Academic institutions and research teams from developed countries, particularly the United States, have dominated the discourse, steering the primary research directions over the past decade. Notably, competency-based education, inter-professional education, and online learning have emerged as the three most prominent pedagogical approaches. These dominant models reflect the convergence of talent development, interdisciplinary synergy, and digital transformation in global health education, offering transformative potential while confronting implementation challenges and equity gaps.

Over the past decade, there has been a significant increase in research on global health curriculum teaching models, primarily led by North American and European countries. From 2014 to 2024, studies in the field have shown a consistent upward trend, particularly accelerated by the COVID-19 pandemic, which underscored the urgent need for training professionals capable of addressing transnational health challenges and further heightened academic institutions’ focus on global health education.^11^ Developed countries, spearheaded by the United States, have established a dominant position in global health education through their early advantages in curriculum development, academic institutional clusters, financial resources, and international collaboration networks. In contrast, developing countries often lack equivalent resources and capacity-building mechanisms, limiting their ability to effectively contribute to or benefit from global health education initiatives.^27^ This dominance by Global North countries highlights significant inequalities in global health education. Research indicates that the predominance of developed countries in global health research has led to a curriculum design and journal review process centered on Global North knowledge systems. However, it is important to note that many global health educational programs, regardless of geographical location, are increasingly emphasizing the integration of diverse cultural perspectives, cultural humility, and intercultural communication skills. These programs aim to amplify indigenous voices and foster a more inclusive approach to global health education, even as curriculum development remains shaped by regional priorities and workforce needs.

Current paradigms of North-South collaboration in global health education reveal fundamental inequities that perpetuate structural imbalances despite surface-level partnerships. In academic publications, many curriculum designs reflect collaborative efforts between the Global North and Global South. However, over half of these collaborations take the form of international field placements, where medical students from HICs are sent to Global South nations for on-site clinical training. This practice raises concerns about potential educational neocolonialism in global health. Host institutions in the Global South often face diverted institutional priorities, increased consumption of material resources, expanded supervisory burdens, and a lack of reciprocal opportunities.^28^ Another critical issue is that, despite incorporating North-South collaboration in curriculum design, many studies still feature a predominance of scholars from HICs as co-authors, while authors from Global South countries often are either underrepresented or excluded from the co-authorship list. This marginalization of contributions from Global South partners reflects a broader pattern of unequal representation in global health publications, even when these partners are involved in the research.^29^ This undermines equitable representation in academic discourse and fails to reflect genuine partnership in program evaluation and feedback. The path to genuine equity requires transforming the entire research-education continuum—from curriculum development to publication practices—through sustained structural reform and power-sharing mechanisms. Many medical schools in the Global North that offer international medical electives are now reforming global health curricula by incorporating pre-departure training, recruiting local faculty, and establishing long-term inter-institutional collaborations as key components.^30^, ^31^

Competency-based education principles have been widely integrated into various global health curriculum teaching models, emerging as a core methodology in global health education. The complexity of global health issues demands that professionals possess the capacity to integrate multidisciplinary knowledge and coordinate diverse resources, a need that competency-based teaching effectively addresses. Global health competencies, defined as a set of interprofessional skills and knowledge, guide educational programs in preparing trainees from various disciplines to tackle complex global health challenges effectively.^32^ Many countries and institutions are now developing frameworks and indicators for global health competencies, driving significant transformations and innovations in global health curricula and teaching models. For instance, the Association of Schools of Public Health (ASPH) established the first Global Health Competency Model 1.1, encompassing seven key domains—Capacity Strengthening, Collaborating and Partnering, Ethical Reasoning and Professional Practice, Health Equity and Social Justice, Program Management, Socio-cultural and Political Awareness, and Strategic Analysis—which has become a key reference standard for public health education worldwide.^33^ Subsequently, CUGH developed a new global health competency framework, referenced in the highly cited study “Identifying Inter-professional Global Health Competencies for 21st-Century Health Professionals”, expanding both the core domains and the applicability to interdisciplinary learners globally.^34^ The current trends in global health education pedagogy align closely with competency-based training requirements. Teaching methodologies such as experiential learning, community-engaged learning, and project-based learning are all primarily designed to enhance students’ multidimensional competencies in global health. Simultaneously, these approaches effectively operationalize constructivist theory and transformative pedagogies by immersing learners in authentic problem-solving and peer-collaboration scenarios.^35^, ^36^

However, these representative frameworks for global health competency have been challenged by criticism such as ambiguous conceptual definitions and inappropriate evaluation metrics, raising questions about their applicability in LMICs and resource-limited settings.^37^ Scholars argue that the development of global health competencies primarily leading by North America countries often fails to adequately incorporate input from host-country health professionals or consider local health contexts. Thus, there is a pressing need for LMICs to develop adapted versions of existing competency models, tailored to their specific workforce training needs. Such contextualization would enhance the relevance and impact of global health education in diverse settings.

The Global Health Learning Objectives (GHLOs), first introduced by the CUGH in 2020 and expanded to include planetary health education in its 2024 version, equip learners at any level of tertiary education—undergraduate or postgraduate—with the foundational understanding of the world required to effectively translate knowledge into skilled applied practice.^20^, ^38^ These objectives cover essential knowledge domains such as the values and history of global health, the health impacts of globalization, socioeconomic determinants, environmental interconnections, ethics, health systems, governance, epidemiology, interventions, and policy evaluation. Compared to global health competencies, which are practice-oriented and often vary across disciplines, the GHLOs are knowledge-based and aim to establish a universal intellectual foundation. This makes them potentially more broadly applicable across diverse academic and professional contexts, as they provide the essential conceptual groundwork necessary before learners can develop specialized skills. However, since this framework is relatively new, its integration into global health education remains limited thus far.

Interdisciplinary integration and cross-cultural trend are also key features in the methodology of global health curricula. On the one hand, the concurrent occurrence of the COVID-19 pandemic and advancing research in ecological and climate systems has fostered the development of innovative paradigms in global health, with “One Health”, “Climate Change”, and “Planetary Health” emerging as pivotal concepts. According to World Health Organization (WHO), One Health is an integrated, unifying approach that seeks to sustainably balance and optimize the health of people, animals, and ecosystems. This approach recognizes the close interconnections and interdependencies among human health, the health of domestic and wild animals, plant health, and the broader environment.^39^ Planetary health is a cross-disciplinary field that has emerged in the last decade. It examines how human-driven changes to natural systems—such as climate change, biodiversity loss, and global pollution—are increasingly affecting human health. These alterations in air, water, soil, and land use are contributing to rising disease burdens and impacting all aspects of human well-being.^40^ These interdisciplinary teaching models have significantly broadened the scope of global health education objects, moving beyond national borders to include cross-species considerations.^41^ This expansion has successfully incorporated environmental determinants into our core focus areas, as well as allowing humanity to conceptualize global health more holistically, embracing fundamental principles like health equity and the “Health for All” initiative. Studies found that inter-professional education enhances both intra-disciplinary and interdisciplinary communication, improving patient health as a whole while also shaping professional identities.^42^ However, this progress simultaneously introduces new challenges in formulating appropriate ethical standards and competency frameworks for global health education.^43^, ^44^ Additionally, a pressing issue requiring further investigation involves the effective integration of emerging paradigms such as geography, foreign languages, communication, diplomacy, sociology, cross-cultural psychology, and international health systems into current global health curricula.^45^ This integration process demands ongoing research and empirical validation to assess the practical effectiveness of interdisciplinary curricula and whether they enhance students' ability to address health challenges.

On the other hand, cross-cultural learning and cultural competency have emerged as critical components of global health education. As global health has evolved into a formal discipline, intercultural competence has increasingly become an essential element in educational curricula. Historically, global health education was often limited to specific international medical electives, where exposure to diverse cultures was more of an added value than a core component. In contrast, modern global health programs now integrate intercultural competence into academic frameworks, recognizing it as a key factor for effective practice, especially as global health services extend beyond traditional medical settings. Cross-cultural understanding is pivotal to global health practice and serves as a critical determinant in achieving global health objectives. The effectiveness of global health interventions in multicultural settings directly depends on the capacity to deconstruct local cultural cognition systems. Key cross-cultural practices include applying local cultural values and mobilizing community religious leaders, among other contextually grounded approaches.^46^ Such learning equips students to navigate diverse cultural contexts and address the complexities of healthcare delivery across cultures. In 2024, Kim et al. examined health issues through multicultural lenses, demonstrating how such an approach enhances students’ cross-cultural communication skills and improves their ability to engage effectively with patients from varied backgrounds.^47^ However, translating cross-cultural principles into tangible knowledge, skills, and attitudes among learners remains challenging. Effective cross-cultural training programs require the integration of innovative educational technologies, rigorous faculty capacity development, and systematic evaluation of curricular effectiveness. Additionally, the implementation of cross-cultural education may encounter institutional resistance to change. Faculty, administrators, and even students may harbor implicit cultural biases, which can undermine their willingness to acknowledge and appreciate diverse values, norms, and perspectives.^48^

The confluence of global health events and technological innovations has also profoundly transformed the educational paradigms of global health curricula, effecting fundamental changes in both pedagogical orientation and educational outcomes. During the COVID-19 pandemic, traditional face-to-face learning opportunities at many higher education institutions were disrupted, marking a pivotal turning point in global medical education.^10^ Online learning, e-learning, and blended learning became key teaching models during the pandemic. Post-pandemic, although the barriers to in-person learning have gradually diminished, blended learning—an innovative teaching model that combines online course materials with face-to-face discussions—has been retained and continues to be a preferred method of instruction.^49^, ^50^ Furthermore, technological advancements, particularly in artificial intelligence, have driven the rapid development of virtual global health courses. These courses are delivered through remote learning tools like Zoom and virtual reality technologies such as remote training.^51^ One significant advantage of virtual education is its ability to simulate complex global health scenarios that might be difficult to replicate in reality. For instance, a team from the Institute of Global Health in Tbilisi, Georgia, developed an augmented reality application that simulates an Ebola outbreak in low-income countries.^52^ Some studies show that virtual education not only helps students engage in realistic global health environments but also enhances their knowledge levels and academic performance.^53^

While technological innovations have undeniably expanded the frontiers of global health education, their integration has simultaneously unveiled persistent tensions between pedagogical promise and equitable implementation. Some educators and students argue that online courses can not satisfactorily replace the hands-on experience gained through traditional face-to-face teaching and clinical practice.^10^ Additionally, from the perspective of health equity, while some institutions in HICs have the technological and financial capacity to shift their educational models to e-learning, educational institutions in LMICs may struggle to fully implement new solutions due to barriers such as slow and unstable internet bandwidth, limited access to computer facilities, and insufficient funding for developing online platforms.^54^, ^55^, ^56^ This technological disparity raises critical concerns about the impact of innovation on existing global health education frameworks. The technological leadership of developed nations in educational innovation may inadvertently exacerbate existing inequalities, potentially creating new forms of educational neocolonialism.^61^ Consequently, as global health education evolves, careful consideration must be given to the ethical implications of technological integration, ensuring equitable access and avoiding the reinforcement of structural disparities.

In the future, the advancement of global health education must prioritize a shift toward a more equitable, inclusive, and contextually grounded paradigm. First, breaking the dominance of HICs and accelerating the decolonization of global health education is imperative. This involves supporting LMICs in building their own education systems, not only by increasing their participation in curriculum development but also by empowering them to contribute meaningfully to academic discourse and research leadership. Second, while competency-based education remains a cornerstone, it must be adapted through culturally specific approaches tailored to the unique sociopolitical and healthcare contexts of each country. Beyond framework development, there is a pressing need for robust mechanisms to assess the applicability and effectiveness of these competencies in diverse settings. Finally, as technology continues to reshape global health pedagogy, it is essential to ensure equitable access and delivery in LMICs. Technological innovations should be implemented alongside careful consideration of digital infrastructure disparities and ethical dimensions, including data security, informed consent, and the risk of educational neocolonialism.

The following re--ations (Box 1**: Re--ations for Stakeholders**) offer a strategic roadmap for educators, researchers, and policymakers to dismantle structural power imbalances and foster a globally representative workforce equipped to address interconnected health challenges. By practicing these re--ations, future global health education can move toward a model that is not only technologically advanced and pedagogically effective but also culturally responsive and socially just.

Box 1 **Re--ations for Stakeholders.**

**Table:** 

**For educators,** • Embed decolonial and locally grounded perspectives into curriculum design, with meaningful LMIC engagement. • Adapt competency-based training and technology-enhanced learning to local contexts, ensuring equitable access and ethical safeguards. **For researchers,** • Strengthen equitable Global North-South research partnerships by promoting fair authorship practices and meaningful LMIC contributions to academic discourse. • Develop and validate context-specific competency frameworks and evaluation tools to assess educational effectiveness across settings. **For policymakers and institutional leaders,** • Invest in sustainable capacity-building for global health education system in LMICs. • Reduce structural and digital inequities by supporting reciprocal partnerships, governance mechanisms, and ethical standards for innovation.

## Strengths and Limitations

This bibliometric analysis offers a comprehensive, multi-dimensional understanding of global health curriculum teaching models over the past decade. The study’s strengths lie in its broad, systematic approach to data collection, utilizing a global perspective and a well-structured search strategy within the WoSCC database. The use of robust bibliometric analysis methods, including annual publication analysis, co-occurring national, institutional, and author analysis, journal and literature co-citation, keyword co-occurrence, and burst detection, enables an in-depth exploration of trends, emerging themes, and influential studies in this field. These methodologies provide valuable insights into the evolution of global health education and its current state.

However, it is essential to acknowledge the limitations of this study. Firstly, to comply with the data format for bibliometric tools in CiteSpace, the study's selection was limited to English-language papers published in the WoSCC database, which may introduce potential publication bias. Future research should consider incorporating studies from multiple databases and languages to provide a more comprehensive and evidence-based summary of global health curriculum teaching models. Second, co-citation analysis primarily reflects associations within internationally prominent journals and may underestimate the contributions of regional journals. Third, the analytical methods were limited to descriptive statistics, such as frequency counts and simple clustering. Consequently, we were unable to perform more sophisticated analyses related to authors’ qualifications and educational backgrounds, resulting in the loss of critical insights regarding learners, educators, and researchers in the field of global health education. Future studies could utilize more advanced software or research methods to constructs multidimensional relationship matrices and explore further information.

## Conclusions

This study systematically assesses the current status, hot topics, and trends in global health curriculum teaching models. Over the past decade, research in this field has significantly increased, primarily driven by North American and European countries, although notable disparities persist. Competency-based education has emerged as a core pedagogical strategy, while interdisciplinary integration and cross-cultural learning have become central features of global health curricula. Concurrently, global health events and technological innovations continue to shape the evolution of teaching models, presenting both opportunities and challenges. As the field progresses, global health education should incorporate culturally specific frameworks, enhance local capacity in LMICs, and ensure equitable access to emerging technologies. By strategically integrating core competencies with emerging technologies the field can prepare a adaptable, globally connected workforce capable of transforming today's health challenges into sustainable solutions for the future.

## Positionality Statement

Our research team comprises researchers and educators with professional backgrounds in global health education and practice. Members are primarily affiliated with institutions in the *Global South* and have substantial experience collaborating with partners from both the *Global North* and *South*. These lived experiences may to some extent shape our views on global health equity, cooperation, and representation.

To mitigate potential biases, we have implemented several methodological safeguards throughout our research process. During the study design phase, we carefully formulated research questions that address pressing contemporary issues in global health education. All analytical indicators, particularly those related to *Global North-South* collaboration and interprofessional cooperation, were predefined and guided by established frameworks, ensuring that our analysis is transparent and reproducible.

In the screening and data extraction phase, two researchers independently conducted reviews, with any discrepancies resolved through discussion to enhance consistency and minimize subjectivity. Our data analysis utilized a bibliometric approach with *CiteSpace* software, systematically identifying patterns in the existing literature to reduce personal biases. Throughout the interpretation and discussion phases, we maintained an objective perspective, substantiating our conclusions with relevant literature to uphold the scientific rigor and clarity of our findings. This comprehensive approach strengthens the reliability of our understanding of global health issues.

## Ethics approval and consent to participate

Not applicable.

## CRediT authorship contribution statement

BQ conceptualized the study, developed and implemented the software used for analysis, conducted the formal data analysis and data visualization, and drafted the original manuscript. YXL contributed to the conceptualization and methodology design, carried out validation and data curation, participated in the review and editing of the manuscript, and supervised the project administration. BW developed and applied the software tools, conducted the formal data analysis and data visualization, and contributed to drafting the original manuscript. YTL contributed to the data visualization and drafting of the original manuscript. KT provided guidance on the conceptualization and methodological framework, contributed to the review and editing of the manuscript, and supervised the project administration. All authors contributed to and approved the final manuscript.

## Funding

Tsinghua University Teaching Reform - Course Innovation Targeted Support Project (DX02_33 Overseas Practice General Education Course Development).

## Consent for publication

Not applicable.

## Declaration of Competing Interest

The authors declare that they have no known competing financial interests or personal relationships that could have appeared to influence the work reported in this paper.

## Data Availability

The data and materials supporting the findings of this study are available within the previously published literature, as well as in this manuscript, including its tables, figures, and supplementary information. For further inquiries or clarifications, please contact the corresponding author, Prof. Kun TANG.

## Footnotes

Appendix A Supplementary data associated with this article can be found in the online version at doi:10.1016/j.ghrp.2026.04.004.
