# Supplementary material for: A bibliometric analysis of global health curriculum teaching models: Current status, hotspots, and trends in higher education between 2014 and 2024
Source: Glob Health Res Policy. 2026 Apr 27;11(1):59–70. doi: 10.1016/j.ghrp.2026.04.004 (PMC13254568; doi:10.1016/j.ghrp.2026.04.004)
Supplement: Supplementary file 1 — Supplementary material [file mmc1.docx]

**Appendix Table 1. Top 10 Literature with the Most Citation**

| Rank | Title (Frequency, Author, Year) | Journal | JCR Partition | Impact Factor |
| --- | --- | --- | --- | --- |
| 1 | Identifying Inter-professional Global Health Competencies for 21st-Century Health Professionals (20, Jogerst K et al., 2015) | [Annals of Global Health](https://www.letpub.com.cn/index.php?page=journalapp&view=detail&journalid=9688) | Public, environmental & occupational health (Q3) | 1.417 |
| 2 | Gaps in studies of global health education: an empirical literature review (10, Liu Y et al., 2015) | Global Health Action | Public, environmental & occupational health (Q2) | 1.712 |
| 3 | A Review of Global Health Competencies for Postgraduate Public Health Education (10, Sawleshwarkar S et al., 2017) | Frontiers in Public Health | Public, environmental & occupational health (Q2) | 2.031* |
| 4 | [Decolonizing global health education: rethinking institutional partnerships and approaches](https://journals.lww.com/academicmedicine/fulltext/2021/03000/Decolonizing_Global_Health_Education__Rethinking.16.aspx) (10, Eichbaum QG et al., 2020) | Academic Medicine | Education, scientific disciplines (Q1)  Health care sciences & services (Q1) | 6.893 |
| 5 | The future of global health education: training for equity in global health (9, Adams LV et al., 2016) | BMC Medical Education | Education & educational research (Q2)  Education, scientific disciplines (Q2) | 1.572 |

**Appendix Table 1. Top 10 Literature with the Most Citation (Continued)**

| Rank | Title (Frequency, Author, Year) | Journal | JCR Partition | Impact Factor |
| --- | --- | --- | --- | --- |
| 6 | The Emergence of Undergraduate Majors in Global Health: Systematic Review of Programs and Recommendations for Future Directions (8, Drain PK et al., 2017) | The American Journal of Tropical Medicine and Hygiene | Public, environmental & occupational health (Q2)  Tropical medicine (Q2) | 2.549 |
| 7 | Global health education in Germany: an analysis of current capacity, needs and barriers (7, Kaffes I et al., 2016) | BMC Medical Education | Education & educational research (Q2)  Education, scientific disciplines (Q2) | 1.572 |
| 8 | Global health education for medical students: New learning opportunities and strategies (6, Goldner BW et al., 2012) | Medical Teacher | Education, scientific disciplines (Q1)  Health care sciences & services (Q2) | 1.824 |
| 9 | Global health education in the United Kingdom: a review of university undergraduate and postgraduate programmes and courses (6, Harmer A et al., 2015) | Public Health | Public, environmental & occupational health  SSCI (Q2)  SCIE (**Q3)** | 1.566 |
| 10 | Reframing undergraduate medical education in global health: Rationale and key principles from the Bellagio Global Health Education Initiative (6, Peluso MJ et al., 2017) | Medical Teacher | Education, scientific disciplines (Q1)  Health care sciences & services (Q2) | 2.450 |

Notes:

1. JCR Partition, Journal Citation Reports Partition;
2. *: Frontier in Public Health’s JCR Partition and Impact Factor are based on 2018 data, as data for 2017 are not available.
